# Deterministic reflection contrast ellipsometry for thick multilayer two-dimensional heterostructures

**DOI:** 10.1515/nanoph-2023-0753

**Published:** 2024-02-07

**Authors:** Kang Ryeol Lee, JinGyu Youn, SeokJae Yoo

**Affiliations:** Inha University, Incheon, Republic of Korea

**Keywords:** two-dimensional materials, van der Waals materials, ellipsometry, reflection contrast spectroscopy, absorption spectroscopy, permittivity

## Abstract

Optical spectroscopy is a powerful tool for characterizing the properties of two-dimensional (2D) heterostructures. However, extracting the permittivity information of each 2D layer in optically thick heterostructures is challenging because of interference. To accurately measure the optical permittivity of each 2D layer in a heterostructure or on a substrate with a thick insulating spacer, such as oxides, we propose deterministic reflection contrast ellipsometry (DRCE). Our DRCE method has two advantages over conventional techniques. It deterministically measures the optical permittivity of 2D materials using only the measured reflection spectra of the heterostructure, rather than dispersion fitting as in spectroscopic ellipsometry. Additionally, the DRCE is free of excitonic energy errors in reflection-contrast spectroscopy. We believe that DRCE will enable accurate and rapid characterization of 2D materials.

## 1 Introduction

A vertical stack of few-atom-thick two-dimensional (2D) materials, known as a 2D heterostructure, has unique properties, such as Moire excitons [1], Wigner crystal states [2], and superconductivity [3], that are not present in the 2D layers of ingredients or their three-dimensional bulk counterparts [4–13]. Optical spectroscopy has been widely used to characterize the properties of 2D heterostructures because it is versatile, noninvasive, and usually requires simple optical components [14]. Stimulated by the huge interest in 2D heterostructures, various optical spectroscopic techniques have been proposed to measure the optical permittivity, a parameter describing the optical response of 2D materials [15], [16], [17], [18], [19], [20], [21]. Spectroscopic ellipsometry (SE) and reflection contrast (RC) spectroscopy are the most common techniques; however, they have inherent drawbacks in the accurate characterization of 2D materials in optically thick 2D heterostructures, whose interference effect is prominent [14].

SE is a common method for determining the permittivity of a general material [22]. SE measures the change in polarization upon reflection and calculates the optical permittivity that reproduces the experimentally measured spectra using a least-squares fit for the known dispersion models [18]. However, prior knowledge of the electronic structure of the 2D material sample outside the spectral region of interest is required [14], [18], [20]; for example, in the monolayer transition metal dichalcogenides (TMDs), electronic transitions in the ultraviolet frequency region can affect the optical permittivity in the visible frequency region [14], [18], [20]. Because of this limitation, determining the optical permittivity of a 2D material sample using only the measured reflection spectra is difficult.

**Figure 1:**
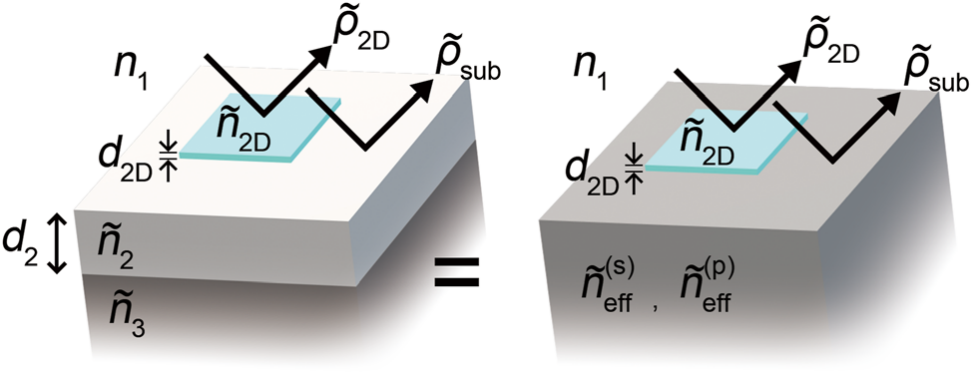
The schematic drawing of DRCE. DRCE characterizes the refractive index of the topmost 2D layer ( ${\tilde{n}}_{2D}$ ) on the multilayers using the measurements of the reflection ratio contrast *δ* = ( ${\tilde{\rho }}_{2D}$ − ${\tilde{\rho }}_{\mathrm{sub}}$ )/ ${\tilde{\rho }}_{\mathrm{sub}}$ between the topmost 2D layer ( ${\tilde{\rho }}_{2D}$ ) and the reduced substrate ( ${\tilde{\rho }}_{\mathrm{sub}}$ ). $\tilde{\rho }={\tilde{r}}^{\left(p\right)}/{\tilde{r}}^{\left(s\right)}$ is the ratio of reflection coefficients ${\tilde{r}}^{\left(p\right)}$ and ${\tilde{r}}^{\left(s\right)}$ for the *p*- and *s*-polarization states. DRCE is based on the effective substrate reduction; reflection by any single layer structure (the left structure in Figure 1) is to that by the reduced substrate of the effective indices (the right structure in Figure 1). The single layer structure is composed of the superstrate (the index *n*_1_), the layer (the index ${\tilde{n}}_{2}$ and the thickness *d*_2_), and the substrate (the index ${\tilde{n}}_{3}$ ).

However, RC spectroscopy is the most prevalent deterministic approach for characterizing 2D materials owing to its simple experimental implementation and deterministic measurement properties [18]. RC spectroscopy measures the reflectance *R* of a 2D sample and its substrate at a normal angle of incidence, and their difference directly indicates the absorption of the 2D sample, that is, the imaginary part of the permittivity, in a deterministic manner [14]. However, RC spectroscopy has two drawbacks: (i) RC spectroscopy measures only the imaginary part of the permittivity if the substrate is nonabsorptive, whereas the real part cannot be obtained directly from the measured spectra. (ii) RC spectroscopy fails when the effects of multiple reflections play an important role in thick optical systems or when the substrate is absorptive [14]. For example, the imaginary part of the permittivity of a 2D material is measured inaccurately when its substrate has an oxide spacer, for example, a SiO_2_/Si substrate, which is typically used to enhance the visibility of atomically thin 2D materials [23], [24], [25], [26], [27], [28], [29]. This drawback of RC spectroscopy is critical for the accuracy of the exciton energy in the permittivity measurement of 2D semiconductors, for example, TMDs, as shown in Figure 1(b). Therefore, RC spectroscopy cannot be applied to thick multilayer 2D heterostructures.

In this study, we propose deterministic reflection contrast ellipsometry (DRCE) to measure the optical permittivity of 2D material layers in an optically thick heterostructure. DRCE measures the changes in polarization upon reflection at each interface in an optically thick heterostructure and algorithmically determines the permittivity using only the measured spectra of the polarization changes. The experimental implementation of the DRCE is also straightforward because it requires simple optics for reflection measurement at an oblique angle of incidence with a few polarization gadgets, such as polarizers and polarization compensators. We theoretically and experimentally demonstrated the validity of a DRCE using 2D TMDs, including monolayer MoS_2_ and WSe_2_ on a Si substrate with a thick SiO_2_ spacer. Our proposed DRCE technique can promote experimental efforts to discover new physics in 2D heterostructures and develop 2D optoelectronic applications.

## 2 Results

### 2.1 Summary of our approach

Figure 1 shows the concept of DRCE. DRCE aims to measure the complex refractive index ${\tilde{n}}_{2D}$ of the topmost 2D layer in the multilayer heterostructure. Analytic determination of the index of the topmost layer on the multilayer is in general not straightforward because of the complicated interference effects within the multilayer. To avoid the complexity, we find that the bottom multilayers can be simplified to the reduced substrate with the effective indices ${\tilde{n}}_{\text{eff}}^{\left(p\right)}$ and ${\tilde{n}}_{\text{eff}}^{\left(s\right)}$ for the *p*- and *s*-polarization states of light, respectively. For example, suppose the topmost 2D layer is sitting on an arbitrary layer (#2, e.g. SiO_2_) of the index ${\tilde{n}}_{2}$ and the thickness *d*_2_. The superstrate (#1, e.g. air) and the substrate (#3, e.g. Si) have the index *n*_1_ and ${\tilde{n}}_{3}$ , respectively. Then, the layer (#2) and the substrate (#3) can be simplified to the reduced substrate of the indices ${\tilde{n}}_{\text{eff}}^{\left(p\right)}$ and ${\tilde{n}}_{\text{eff}}^{\left(s\right)}$ .

Using the effective substrate reduction, any optically thick multilayer can be replaced with a single substrate. This enables analytic determination of the index of the topmost layer on the multilayer. DRCE measurement combines conventional ellipsometry and RC spectroscopy. As in conventional ellipsometry, DRCE measures the complex reflection ratio $\tilde{\rho }={\tilde{r}}^{\left(p\right)}/{\tilde{r}}^{\left(s\right)}$ , where ${\tilde{r}}^{\left(p\right)}$ and ${\tilde{r}}^{\left(s\right)}$ denote the reflection coefficient of the *p*- and *s*-polarization states, respectively. Similar to RC spectroscopy, DRCE measures the reflection ratio contrast *δ* = ( ${\tilde{\rho }}_{2D}$ − ${\tilde{\rho }}_{\mathrm{sub}}$ )/ ${\tilde{\rho }}_{\mathrm{sub}}$ between the topmost 2D layer ( ${\tilde{\rho }}_{2D}$ ) and the reduced substrate ( ${\tilde{\rho }}_{\mathrm{sub}}$ ) instead of the reflection contrast. Then, we can analytically determine the complex refractive index ${\tilde{n}}_{2D}$ of the topmost 2D layer in the multilayer heterostructure. Details on the effective substrate reduction and DRCE are described in the following subsections.

### 2.2 Effective substrate reduction

The proposed DRCE method is based on effective substrate reduction; light reflection by a single layer on a substrate is equivalent to that of a reduced substrate whose effective refractive index ${\tilde{n}}_{\text{eff}}$ is different from that of the original layer or substrate (refer to Supplementary Materials for the proof). In other words, the Airy formula for a single layer on a substrate is equivalent to the Fresnel formula for an interface between two semi-infinite media if the effective index is adequately determined.

**Figure 2:**
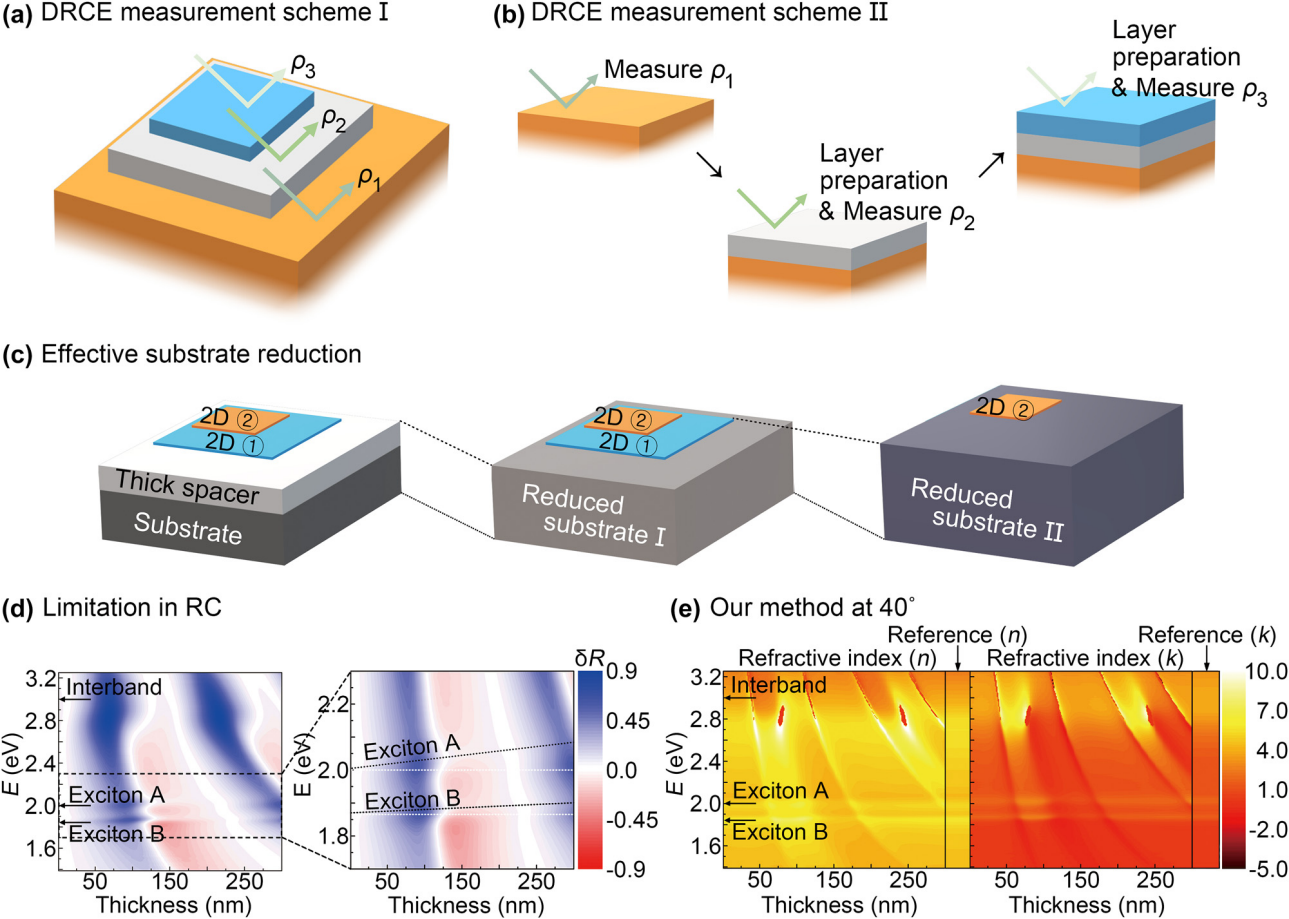
Two experimental schemes for deterministic reflection contrast ellipsometry (DRCE): (a) Scheme I: A staircase multilayer structure; reflection ratio ( $\tilde{\rho }$ ) measurements are performed at the interface of each layer and air. (b) Scheme II: A general multilayer structure; reflection ratio measurements are performed before the next layer preparation. (c) Schematic drawing of the subsequent effective substrate reduction for DRCE. (d) Reflection contrast (RC) spectra *δR* of monolayer MoS_2_ on the SiO_2_/Si substrate is plotted as a function of oxide thickness. Exciton energies are depicted in the black dashed line in the magnified inset. Apparent peaks in RC spectra are shifted as oxide thickness varies, resulting in inaccurate measurement of exciton energies using RC spectroscopy. (e) The complex refractive index $\tilde{n}=n+ik$ of the same monolayer MoS_2_ on the SiO_2_/Si substrate characterized by DRCE at the angle of incidence of 40°. The reference refractive index is obtained from the literature [20], and it is plotted in the left and right sides of the plots. Exciton energies are not shifted in our method although some errors in their magnitudes depend on the oxide thickness. Figure 1(d) and (e) show theoretical calculations.

This equivalence allows the reduction of a *N* layer system to a (*N* − 1) layer system if we know all the s-wave ( ${\tilde{r}}^{\left(s\right)}$ ) and p-wave ( ${\tilde{r}}^{\left(p\right)}$ ) reflections at the interface between each layer and the superstrate (e.g., air). Experimentally, this condition for DRCE can be achieved in two ways: (i) a multilayer system with a staircase structure, and each layer has an exposed air/layer interface (Figure 2(a)); many lab-scale 2D heterostructures fall into this category because the sizes of mechanically exfoliated and chemically grown 2D flakes are typically different from each other. (ii) If the staircase multilayer structure cannot be achieved, reflection and layer transfer are alternately performed so that we can determine the reflection at each layer/air interface (Figure 2(b)). 2D heterostructures prepared by the subsequent chemical deposition of layers fall into this category. We note that the theoretical values of the reflection coefficients ${\tilde{r}}^{\left(s\right)}$ and ${\tilde{r}}^{\left(p\right)}$ can be used in DRCE if the permittivity of several ingredient layers is known and their optical characterization is not of interest. For example, for the characterization of a monolayer TMD on a SiO_2_/Si substrate, we do not need to measure reflections at the interfaces of air/Si and air/SiO_2_/Si substrates because we can calculate the reflection coefficients at such interfaces using the permittivity reported in the literature. We note that two measurement schemes in Figure 2(a) and (b) are applicable to heterostructures with no or weak interlayer coupling because strong interlayer coupling can change the permittivity of each 2D layer.

Subsequent reduction of the effective substrate yielded a single top layer on the reduced substrate (Figure 2(c)). This suggests that the complicated ellipsometry problem for the N-layer system can always be reduced to a simple RC ellipsometry problem for a single-layer system if we can measure the reflections of the s-wave ( ${\tilde{r}}^{\left(s\right)}$ ) and p-wave ( ${\tilde{r}}^{\left(p\right)}$ ), that is, the complex reflection ratio $\tilde{\rho }={\tilde{r}}^{\left(p\right)}/{\tilde{r}}^{\left(s\right)}$ , at the interface between each layer and the superstrate, as shown in Figure 1(c). Note that the measurement of $\tilde{\rho }$ can be achieved using commercial ellipsometers or a home-made setup, such as a rotating compensator ellipsometer (RCE) [30].

Analytically, the effective refractive indices of the reduced substrate replacing the single layer and its substrate are expressed as follows (see Supplementary Materials for details on the derivation),

(1) $$\begin{array}{ll} {\left({\tilde{n}}_{\text{eff}}^{\left(p\right)}\right)}^{2} & =\frac{1}{2cos^{2}{\tilde{\theta }}_{2}}\left\{{{\tilde{n}}_{2}}^{2}{\left({\tilde{\beta }}^{\left(p\right)}\right)}^{2}\right.\\ & \;\left.\pm \sqrt{{{\tilde{n}}_{2}}^{4}{\left({\tilde{\beta }}^{\left(p\right)}\right)}^{4}-4{n_{1}}^{2}{{\tilde{n}}_{2}}^{2}{\left({\tilde{\beta }}^{\left(p\right)}\right)}^{2}sin^{2}\theta_{1}cos^{2}{\tilde{\theta }}_{2}}\right\}, \end{array}$$

(2) $${\left({\tilde{n}}_{\text{eff}}^{\left(s\right)}\right)}^{2}={n_{1}}^{2}sin^{2}\theta_{1}+\left({{\tilde{n}}_{2}}^{2}-{n_{1}}^{2}sin^{2}\theta_{1}\right){\left({\tilde{\beta }}^{\left(s\right)}\right)}^{2},$$

for the *p*- and *s*-polarizations of light, respectively. Notations in Eqs. (1) and (2) follow Figure 1. Plus-minus sign in Eq. (1) can be determined by continuous and nonvanishing values in ${\tilde{n}}_{\text{eff}}^{\left(p\right)}$ because null values in the effective indices result in the failure of DRCE (see Supporting Information for details). The single layer has an index of ${\tilde{n}}_{2}$ and a thickness of *d*_2_, while the indices of the semi-infinite superstrate and substrate are *n*_1_ and ${\tilde{n}}_{3}$ , respectively. *θ*_1_ and ${\tilde{\theta }}_{2}=sin^{-1}\left[\left(n_{1}/{\tilde{n}}_{2}\right)\sin\theta_{1}\right]$ denote the angles of incidence and refraction in the layer, respectively. In Eq. (2), function $\tilde{\beta }$ is expressed as follows:

(3) $${\tilde{\beta }}^{\left(p,s\right)}=\frac{1-{\tilde{r}}_{23}^{\left(p,s\right)}e^{2i{\tilde{\phi }}_{2}}}{1+{\tilde{r}}_{23}^{\left(p,s\right)}e^{2i{\tilde{\phi }}_{2}}},$$

where ${\tilde{\phi }}_{2}={\tilde{k}}_{2}d_{2}\cos{\tilde{\theta }}_{2}$ is the optical path length inside the layer whose thickness is *d*_2_ and wavenumber is ${\tilde{k}}_{2}$ , and the Fresnel equation determines ${{\tilde{r}}_{23}}^{\left(p\right)}=\left({\tilde{n}}_{2}\cos{\tilde{\theta }}_{3}-{\tilde{n}}_{3}\cos{\tilde{\theta }}_{2}\right)/\left({\tilde{n}}_{2}\cos{\tilde{\theta }}_{3}+{\tilde{n}}_{3}\cos{\tilde{\theta }}_{2}\right)$ and ${{\tilde{r}}_{23}}^{\left(s\right)}=\left({\tilde{n}}_{2}\cos{\tilde{\theta }}_{2}-{\tilde{n}}_{3}\cos{\tilde{\theta }}_{3}\right)/\left({\tilde{n}}_{2}\cos{\tilde{\theta }}_{2}+{\tilde{n}}_{3}\cos{\tilde{\theta }}_{3}\right)$ in Eq. (3). ${\tilde{\theta }}_{3}=sin^{-1}\left[\left(n_{1}/{\tilde{n}}_{3}\right)\sin{\tilde{\theta }}_{3}\right]$ is the angle of refraction in the substrate. Note that Eqs. (1) and (2) are reduced to our previous result in limit of the normal angle of incidence [31].

### 2.3 Deterministic reflection contrast ellipsometry (DRCE)

By the effective substrate reduction, any multilayer system can be reduced to the reduced substrate whose effective indices are given by Eqs. (1) and (2). Let a 2D material layer of index ${\tilde{n}}_{2D}$ and thickness *d*_*2D*_ is sitting on the reduced substrate. Once we can measure the reflection ratio contrast *δ* = ( ${\tilde{\rho }}_{2D}$ − ${\tilde{\rho }}_{\mathrm{sub}}$ )/ ${\tilde{\rho }}_{\mathrm{sub}}$ between the top 2D layer ( ${\tilde{\rho }}_{2D}$ ) and the reduced substrate ( ${\tilde{\rho }}_{\mathrm{sub}}$ ), the refractive index can be obtained as follows:

(4) $$\begin{array}{ll} {\left({\tilde{n}}_{2D}\right)}^{2} & =\frac{1}{2B}\left\{-A+B{n_{1}}^{2}-\frac{\delta }{\alpha }\right.\\ & \;\left.\pm \sqrt{4AB{n_{1}}^{2}+{\left(A-B{n_{1}}^{2}+\frac{\delta }{\alpha }\right)}^{2}}\right\}, \end{array}$$

where the functions *A*, *B*, and *α* are defined as follows:

(5) $$A=-2{\left({\tilde{n}}_{\text{eff}}^{\left(p\right)}\right)}^{4}\left\{{n_{1}}^{2}-{\left({\tilde{n}}_{\text{eff}}^{\left(s\right)}\right)}^{2}\right\}sin^{2}\theta_{1},$$

(6) $$\begin{array}{ll} B & ={\left({\tilde{n}}_{\text{eff}}^{\left(p\right)}\right)}^{4}+{n_{1}}^{2}{\left({\tilde{n}}_{\text{eff}}^{\left(s\right)}\right)}^{2}-2{\left({\tilde{n}}_{\text{eff}}^{\left(s\right)}\right)}^{2}{\left({\tilde{n}}_{\text{eff}}^{\left(p\right)}\right)}^{2}\\ & \;+\left\{{\left({\tilde{n}}_{\text{eff}}^{\left(p\right)}\right)}^{4}-{n_{1}}^{2}{\left({\tilde{n}}_{\text{eff}}^{\left(s\right)}\right)}^{2}\right\}cos2\theta_{1}, \end{array}$$

(7) $$\alpha =\frac{2\pi }{\lambda }\frac{id_{2D}n_{1}\sec\theta_{1}}{\left\{{n_{1}}^{2}-{\left({\tilde{n}}_{\text{eff}}^{\left(s\right)}\right)}^{2}\right\}\left\{{\left({\tilde{n}}_{\text{eff}}^{\left(s\right)}\right)}^{4}-{n_{1}}^{2}{\left({\tilde{n}}_{\text{eff}}^{\left(p\right)}\right)}^{2}sec^{2}\theta_{1}+{n_{1}}^{4}tan^{2}\theta_{1}\right\}},$$

where *λ* is the wavelength of light. Notations in Eqs. (4)–(7) follow Figure 1. Eq. (4) is a key result of this study. Note that *n*_1_ is the index of the superstrate in Eqs. (4)–(7). All variables in Eqs. (4)–(7), except for the experimentally measured *δ*, are known for a given sample. It is also noteworthy that Eq. (4) is obtained by the first-order expansion of *δ* in a function of the optical path length ${\tilde{\phi }}_{2D}={\tilde{k}}_{2D}d_{2D}\cos{\tilde{\theta }}_{2D}$ , and the second-order expansion can increase the accuracy of DRCE [15].

In DRCE, the angle of incidence should be carefully chosen because Eq. (4) is singular if ${\tilde{n}}_{\text{eff}}^{\left(p\right)}$ and ${\tilde{n}}_{\text{eff}}^{\left(s\right)}$ become 0 or ±1. We find that the singularity can occur when light interferes destructively. The destructive interference condition is given by $\left(2m+1\right)\left(\lambda /4\right)=n_{i}d_{i}\cos\theta_{i}$ with the refractive index *n*_*i*_, the thickness *d*_i_, the integer *m*, and the angle of propagation in an arbitrary *i*th layer of the multilayer heterostructure. Therefore, large angle of incidence can push the spectral region of the interference-induced errors to the short wavelengths. Note that one should minimize errors in each measurement step (Figure 2(a)) for the reflection ratio contrast *δ* because errors can be accumulated, and it results in inaccurate results of the refractive indices.

### 2.4 Experiment and simulation results

**Figure 3:**
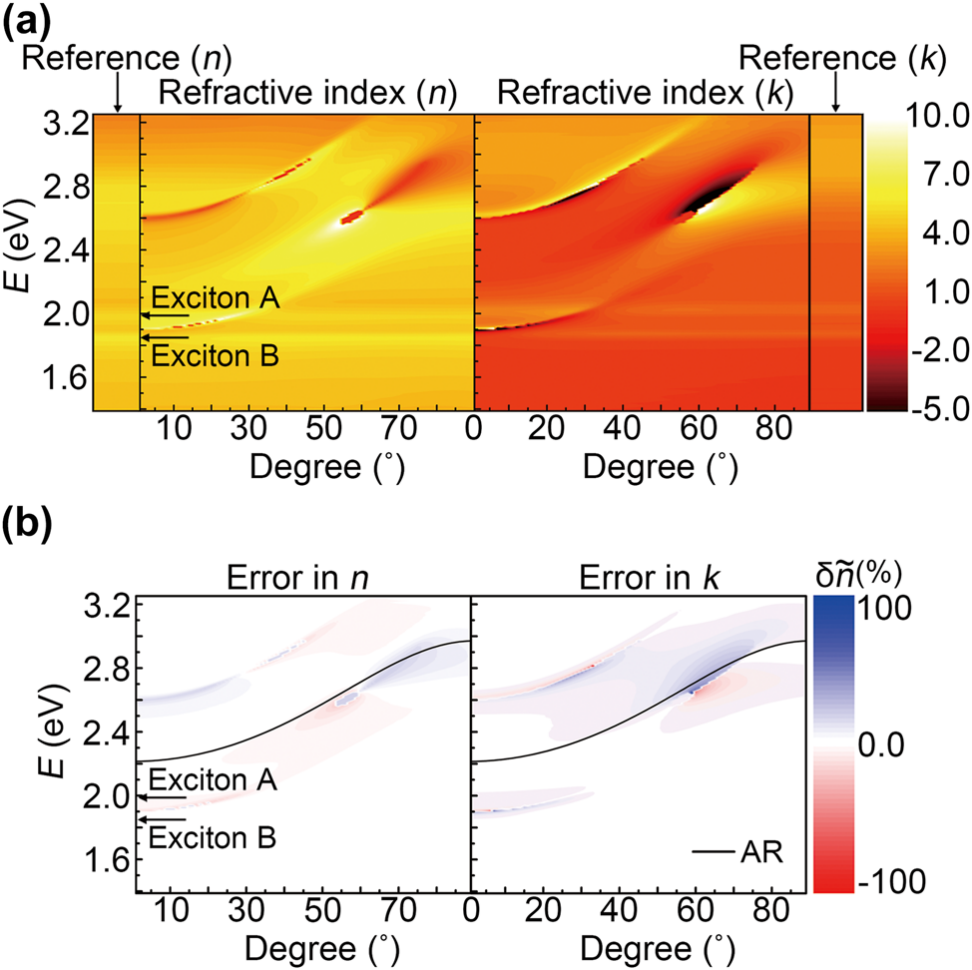
Simulation for the DRCE measurement of the refractive index of monolayer MoS_2: (a) Refractive index $\tilde{n}=n+ik$ of monolayer MoS_2_ characterized by DRCE with different angles of incidence. Monolayer MoS_2_ is sitting on the SiO_2_/Si substrate with oxide thickness of 280 nm. The real (*n*_*ref*_) and imaginary parts (*k*_*ref*_) of the reference refractive index [20] ( ${\tilde{n}}_{\mathrm{ref}}=n_{\mathrm{ref}}+ik_{\mathrm{ref}}$ ) are plotted in the left and right sides, respectively. Exciton energies are displayed by black arrows. (b) Corresponding errors of DRCE for the refractive index ( $\delta n_{\mathrm{ref}}=\left(n_{\mathrm{ref}}-n\right)/n_{\mathrm{ref}}$ and $\delta k_{\mathrm{ref}}=\left(k_{\mathrm{ref}}-k\right)/k_{\mathrm{ref}}$ ). Black lines in (b) are destructive interference condition of the oxide layer.

To verify the permittivity characterization using Eq. (4) for a given reflection-ratio contrast *δ*, we theoretically simulated the DRCE of monolayer MoS_2_ on a SiO_2_/Si substrate with an oxide thickness of 280 nm. In our previous study, we calculated the reflection ratio contrast *δ* using the transfer matrix technique [32] and tabulated the permittivity of monolayer MoS_2_. We use the monolayer thickness *d*_2D_ = 0.65 nm in the following [20]. As illustrated in Figure 3, we compare the permittivity obtained by DRCE at various angles of incidence (color profiles) with the reference permittivity [20] (insets on the left and right sides). First, we identified that the positions of the excitonic peaks remained nearly constant and were the same as the reference permittivity for all angles of incidence. Second, the DRCE results for the permittivity became more accurate over a broad energy range as the angle of incidence increased. To quantify the accuracy of DRCE, we plot its error, ( $\delta \tilde{n}=\left({\tilde{n}}_{\mathrm{ref}}-\tilde{n}\right)/{\tilde{n}}_{\mathrm{ref}}$ ), in Figure 3(b). The error shifted to a higher-energy region as the angle of incidence increased, as shown in Figure 3(a). The error region follows the destructive interference induced by the oxide layer (black lines in Figure 3(b)).

**Figure 4:**
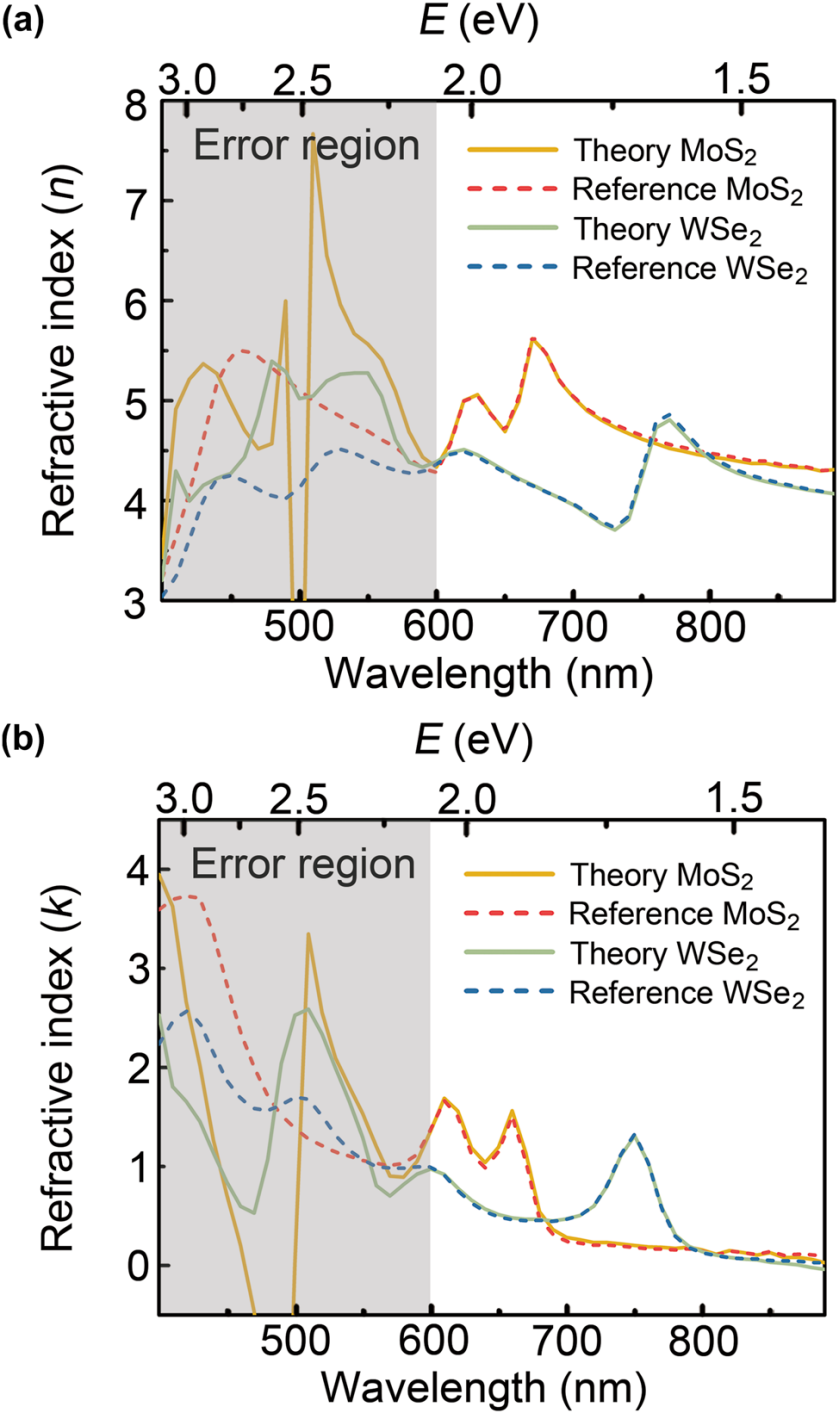
DRCE characterization of the refractive index of each 2D monolayer TMD in the heterostructure composed of monolayer MoS_2_/monolayer WSe_2_/280 nm-thick SiO_2_/Si substrate. (a) The real (*n*) and (b) imaginary parts (*k*) of the refractive index (the orange solid line: DRCE result of monolayer MoS_2_, the green solid line: DRCE result of monolayer WSe_2_, the red dashed line: reference value for of monolayer MoS_2_, and blue dashed line: reference value for of monolayer WSe_2_). In Figure 3, the angle of incidence is fixed at 50°. Reference refractive indices of monolayer MoS_2_ and WSe_2_ are obtained from our previous study [20].

In addition, we simulated the DRCE of a 2D heterostructure composed of a monolayer MoS_2_/monolayer WSe_2_/280 nm-thick SiO_2_/Si substrate in Figure 4. The refractive index of each monolayer TMDs can be obtained sequentially by the reflection ratio contrast *δ* at each layer/air interface. (i) Using the reflection ratio contrast *δ* between the SiO_2_ layer and the substrate, oxide layer and substrate are replaced with the first reduced substrate. (ii) Using the reflection ratio contrast *δ* between the monolayer WSe_2_ and SiO_2_/Si substrate (i.e., the first reduced substrate), we can obtain the refractive index of the monolayer WSe_2_ using Eq. (4). Moreover, the reflection ratio contrast *δ* yields the second reduced substrate. (iii) Finally, using the reflection ratio contrast *δ* between monolayer MoS_2_ and the monolayer WSe_2_/SiO_2_/Si substrate (i.e., the second reduced substrate), we can obtain the refractive index of the monolayer MoS_2_ by Eq. (4). This procedure is illustrated in Figure 2(a). Figure 4(a) and (b) show the refractive indices of the two monolayers at an incident angle of 50°. As mentioned in the discussion of Figure 3, the errors are prominent in the short-wavelength region (i.e., the high-energy region). In the long-wavelength region, the refractive index can be accurately measured using the DRCE. We also performed DRCE measurements at higher angles (Figure S2 in the Supplementary Material). Higher angle measurements show that the error region moves to the shorter wavelengths, as also shown in Figure 3(b). We emphasize that multilayered 2D heterostructures with more than two layers can be characterized in the same manner, that is, the subsequent reduction of the reduced substrate and DRCE.

**Figure 5:**
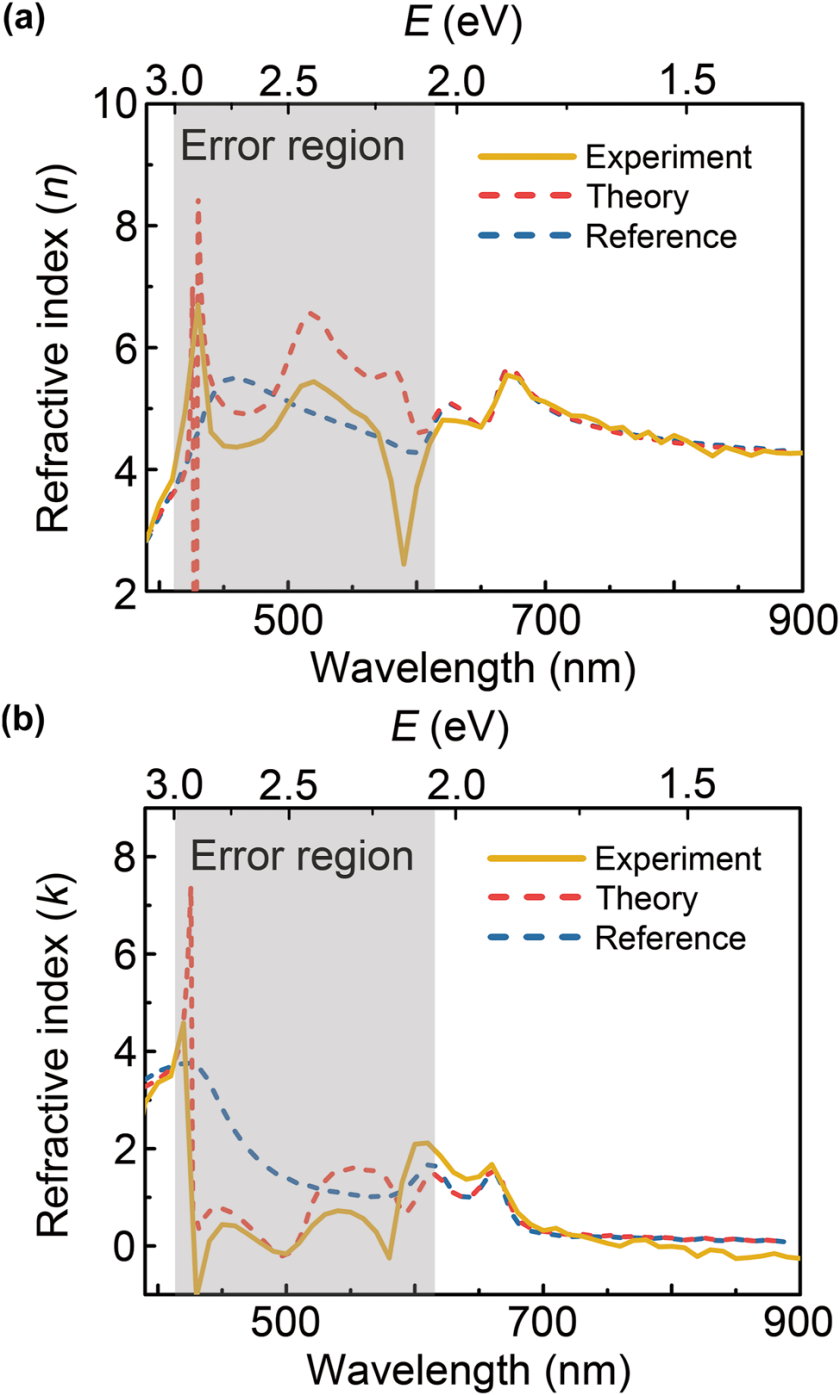
DRCE experiment for monolayer MoS_2_ in the heterostructure composed of CVD-grown monolayer MoS_2_/280 nm-thick SiO_2_/Si substrate. (a) The real (*n*) and (b) imaginary parts (*k*) of refractive index characterized at the incident angle of 40°. (The solid orange line: DRCE experiment results, the dashed red line: DRCE theoretical simulation results, and the dashed blue line: reference refractive index of monolayer MoS_2_ obtained from our previous study [20]).

In Figure 5, we performed a DRCE experiment on a chemical vapor deposition (CVD)-grown monolayer MoS_2_ on a SiO_2_/Si substrate with an oxide thickness of 280 nm (purchased from SixCarbon), which has the same structure as the theoretical simulation of DECR shown in Figure 3. The reflection ratio $\tilde{\rho }$ in the visible wavelength region was measured using a commercial spectroscopic ellipsometer (J.A. Woollam, V-VASE) at an angle of incidence of 40°. This angle of incidence was chosen because the peaks of the A and B excitons were clearly identified in the theoretical simulation shown in Figure 3. As shown in Figure 5, the refractive indices obtained by DRCE (solid orange line), theoretical simulation (dashed red line), and literature values [20] (dashed blue line) demonstrated good agreement, except in the high-energy error region. Our experiments confirmed the validity of DRCE for 2D heterostructures.

## 3 Conclusions

We addressed the issues in conventional optical techniques, such as RC spectroscopy and SE, to characterize the permittivity of 2D material layers in optically thick heterostructures. The conventional techniques had some limitations, such as (i) nondeterministic permittivity measurements requiring dispersion fitting (in SE [18]), (ii) partial measurement of the imaginary part of the permittivity (in RC spectroscopy [16]), and (iii) measurement failure on the substrate with a thick oxide spacer layer (in RC spectroscopy and our previous study [20]). We demonstrated that DRCE is free from such limitations in determining the permittivity of each 2D material layer in the heterostructure (see Figure S3 for example to compare our method to conventional methods). We also note that our method can be applied to transparent 2D materials such as graphene and hBN (Figure S4). Our method may enable the rapid and noninvasive optical characterization of 2D material layers in thick heterostructures.

## Supplementary Material

Supplementary Material Details
