# Supplementary material for: Deterministic reflection contrast ellipsometry for thick multilayer two-dimensional heterostructures
Source: Nanophotonics. 2024 Feb 7;13(8):1417–24. doi: 10.1515/nanoph-2023-0753 (PMC11636472; doi:10.1515/nanoph-2023-0753)
Supplement: Supplementary file 1 — Supplementary Material Details [file j_nanoph-2023-0753_suppl_001.pdf]

Supplementary Material:

Deterministic reflection contrast ellipsometry for thick multilayer two-dimensional heterostructures

Kang Ryeol Lee, JinGyu Youn, SeokJae Yoo

Department of Physics, Inha University, Incheon, Korea 22212

1. Details in the effective substrate reduction (Eqs. (1) and (2))

We provide Eqs. (1) and (2), key equations for the effective substrate reduction, in the main text. Here, we explain details in the mathematical derivation. We start from the equivalence of the Fresnel formula (*i.e.* the reflection of an interface) and the Airy formula (*i.e.* the reflection of a layer on a substrate). The Airy formula is given by [1]

$$r^{(p/s)} = \frac{r_{12}^{(p/s)} + r_{23}^{(p/s)} e^{2i\phi}}{1 + r_{12}^{(p/s)} r_{23}^{(p/s)} e^{2i\phi}}, \tag{S1}$$

where the superscript *p* (*s*) denotes the p-polarization (s-polarization) of light. The subscript 1, 2, and 3 denote the superstrate (*e.g.* air), the layer, and the substrate, respectively. $\phi = k_2 d_2 \cos\theta_2$ is the optical path length inside the layer whose thickness is *d*. $r_{ij}^{(p/s)}$ are the Fresnel formulas at the interface between media *i* and *j* (*i*, *j* = 1, 2, and 3), and they are given by

$$r_{ij}^{(p)} = \frac{\left(n_i^2 / k_{ix}\right) - \left(n_j^2 / k_{jx}\right)}{\left(n_i^2 / k_{ix}\right) + \left(n_j^2 / k_{jx}\right)} = \frac{n_i^2 k_{jx} - n_j^2 k_{ix}}{n_i^2 k_{jx} + n_j^2 k_{ix}}, \tag{S2}$$

$$r_{ij}^{(s)} = \frac{k_{ix} - k_{jx}}{k_{ix} + k_{jx}}, \tag{S3}$$

where $n_i = \sqrt{\varepsilon_i}$ and $k_{x,i} = n_i k_0 \cos\theta_i$ are the complex-valued refractive index and the x-component of the wavevector in the *i*-th medium with the vacuum wavenumber $k_0 = 2\pi / \lambda$ and the angle of propagation θ_i. First of all, we consider the Fresnel/Airy equivalence for the p-polarization.

Plugging Eq. (S2) into the Airy formula, Eq. (S1), gives

$$
\begin{aligned}
r^{(p)} &= \frac{r_{12}^{(p)} + r_{23}^{(p)} e^{2i\phi}}{1 + r_{12}^{(p)} r_{23}^{(p)} e^{2i\phi}} \\
&= \frac{\left(n_1^2 k_{2x} - n_2^2 k_{1x}\right)\left(n_2^2 k_{3x} + n_3^2 k_{2x}\right) + \left(n_2^2 k_{3x} - n_3^2 k_{2x}\right)\left(n_1^2 k_{2x} + n_2^2 k_{1x}\right) e^{2i\phi}}{\left(n_1^2 k_{2x} + n_2^2 k_{1x}\right)\left(n_2^2 k_{3x} + n_3^2 k_{2x}\right) + \left(n_1^2 k_{2x} - n_2^2 k_{1x}\right)\left(n_2^2 k_{3x} - n_3^2 k_{2x}\right) e^{2i\phi}} \\
&= \frac{n_1^2 k_{2x} \left\{ n_2^2 k_{3x}\left(1+e^{2i\phi}\right) + n_3^2 k_{2x}\left(1-e^{2i\phi}\right)\right\} - n_2^2 \left\{ n_2^2 k_{3x}\left(1-e^{2i\phi}\right) + n_3^2 k_{2x}\left(1+e^{2i\phi}\right)\right\} k_{1x}}{n_1^2 k_{2x} \left\{ n_2^2 k_{3x}\left(1+e^{2i\phi}\right) + n_3^2 k_{2x}\left(1-e^{2i\phi}\right)\right\} + n_2^2 \left\{ n_2^2 k_{3x}\left(1-e^{2i\phi}\right) + n_3^2 k_{2x}\left(1+e^{2i\phi}\right)\right\} k_{1x}} \\
&= \frac{\left(n_1^2 / k_{1x}\right) - \dfrac{n_2^2}{k_{2x}} \dfrac{n_2^2 k_{3x}\left(1-e^{2i\phi}\right) + n_3^2 k_{2x}\left(1+e^{2i\phi}\right)}{n_2^2 k_{3x}\left(1+e^{2i\phi}\right) + n_3^2 k_{2x}\left(1-e^{2i\phi}\right)}}{\left(n_1^2 / k_{1x}\right) + \dfrac{n_2^2}{k_{2x}} \dfrac{n_2^2 k_{3x}\left(1-e^{2i\phi}\right) + n_3^2 k_{2x}\left(1+e^{2i\phi}\right)}{n_2^2 k_{3x}\left(1+e^{2i\phi}\right) + n_3^2 k_{2x}\left(1-e^{2i\phi}\right)}} \\
&= \frac{\left(n_1^2 / k_{1x}\right) - \dfrac{n_2^2}{k_{2x}} \beta^{(p)}\left(n_2, n_3\right)}{\left(n_1^2 / k_{1x}\right) + \dfrac{n_2^2}{k_{2x}} \beta^{(p)}\left(n_2, n_3\right)}
\end{aligned}
\quad , \text{(S4)}
$$

where the last line of Eq. (S4) can be obtained by

$$
\begin{aligned}
\frac{n_2^2}{k_{2x}} \frac{n_2^2 k_{3x}\left(1-e^{2i\phi}\right) + n_3^2 k_{2x}\left(1+e^{2i\phi}\right)}{n_2^2 k_{3x}\left(1+e^{2i\phi}\right) + n_3^2 k_{2x}\left(1-e^{2i\phi}\right)} &= \frac{n_2^2}{k_{2x}} \frac{1 - \dfrac{n_2^2 k_{3x} - n_3^2 k_{2x}}{n_2^2 k_{3x} + n_3^2 k_{2x}} e^{2i\phi}}{1 + \dfrac{n_2^2 k_{3x} - n_3^2 k_{2x}}{n_2^2 k_{3x} + n_3^2 k_{2x}} e^{2i\phi}} \\
&= \frac{n_2^2}{k_{2x}} \frac{1 - r_{23}^{(p)} e^{2i\phi}}{1 + r_{23}^{(p)} e^{2i\phi}} \\
&\equiv \frac{n_2^2}{k_{2x}} \beta^{(p)}\left(n_2, n_3\right)
\end{aligned}
\quad \text{(S5)}
$$

We want the last line of Eq. (S4) to be expressed in the same form of the Fresnel equation,

$$
r^{(p)} = \frac{\left(n_1^2 / k_{1x}\right) - \left\{ \left(n_{eff}^{(p)}\right)^2 / k_{eff,x}^{(p)} \right\}}{\left(n_1^2 / k_{1x}\right) + \left\{ \left(n_{eff}^{(p)}\right)^2 / k_{eff,x}^{(p)} \right\}}, \quad \text{(S6)}
$$

Equivalence of the last line of Eq. (S4) and Eq. (S6) requires

$$
\left(n_{eff}^{(p)}\right)^2 / k_{eff,x}^{(p)} = \frac{n_2^2}{k_{2x}} \beta^{(p)}\left(n_2, n_3\right). \quad \text{(S7)}
$$

Without loss of generality, we are also able to define the effective wavevector component, *i.e.* $k_{eff,x}^{(p)} = n_{eff}^{(p)} k_0 \cos\theta_{eff}^{(p)}$, and the angle of propagation in the effective substrate using the Snell's

law, *i.e.* $\cos\theta_{eff}^{(p)} = \sqrt{1-\left(n_1 / n_{eff}^{(p)}\right)^2 \sin^2\theta_1}$. Then, the left-hand and right-hand side of Eq. (S7) can be written as

$$\left(n_{eff}^{(p)}\right)^2 / k_{eff,x}^{(p)} = \frac{n_{eff}^{(p)}}{k_0\sqrt{1-\left(n_1 / n_{eff}^{(p)}\right)^2 \sin^2\theta_1}}, \tag{S8}$$

$$\frac{n_2^2}{k_{2x}}\beta^{(p)}\left(n_2,n_3\right) = \frac{n_2}{k_0\cos\theta_2}\beta^{(p)}\left(n_2,n_3\right), \tag{S9}$$

respectively. Combining Eqs. (S8) and (S9) yields the quadratic equation for $\left(n_{eff}^{(p)}\right)^2$,

$$\left(n_{eff}^{(p)}\right)^4 \cos^2\theta_2 - n_2^2\left\{\beta^{(p)}\left(n_2,n_3\right)\right\}^2\left(n_{eff}^{(p)}\right)^2 + n_1^2 n_2^2\left\{\beta^{(p)}\left(n_2,n_3\right)\right\}^2\sin^2\theta_1 = 0 \tag{S10}$$

Solving Eq. (S10) for the effective index $\left(n_{eff}^{(p)}\right)^2$, we can obtain Eq. (1) in the main text. Eq. (1) and (S6) show that a problem of light reflection by a layer on a substrate (*i.e.* the Airy formula) can be reduced to that by a single interface between the superstrate and the effective substrate (*i.e.* the Fresnel formula).

Likewise, we can obtain expression for the s-polarization, Eq. (2) in the main text. Plugging Eq. (S3) into Eq. (S1), the Airy formula (Eq. (S1)) becomes

$$\begin{aligned} r^{(s)} &= \frac{r_{12}^{(s)} + r_{23}^{(s)}e^{2i\phi}}{1+r_{12}^{(s)}r_{23}^{(s)}e^{2i\phi}} \\ &= \frac{\left(k_{1x}-k_{2x}\right)\left(k_{2x}+k_{3x}\right)+\left(k_{1x}+k_{2x}\right)\left(k_{2x}-k_{3x}\right)e^{2i\phi}}{\left(k_{1x}+k_{2x}\right)\left(k_{2x}+k_{3x}\right)+\left(k_{1x}-k_{2x}\right)\left(k_{2x}-k_{3x}\right)e^{2i\phi}} \\ &= \frac{k_{1x}\left\{k_{2x}+k_{3x}+\left(k_{2x}-k_{3x}\right)e^{2i\phi}\right\} - k_{2x}\left\{k_{2x}+k_{3x}-\left(k_{2x}-k_{3x}\right)e^{2i\phi}\right\}}{k_{1x}\left\{k_{2x}+k_{3x}+\left(k_{2x}-k_{3x}\right)e^{2i\phi}\right\} + k_{2x}\left\{k_{2x}+k_{3x}-\left(k_{2x}-k_{3x}\right)e^{2i\phi}\right\}} \\ &= \frac{k_{1x} - k_{2x}\dfrac{\left(k_{2x}+k_{3x}\right)-\left(k_{2x}-k_{3x}\right)e^{2i\phi}}{\left(k_{2x}+k_{3x}\right)+\left(k_{2x}-k_{3x}\right)e^{2i\phi}}}{k_{1x} + k_{2x}\dfrac{\left(k_{2x}+k_{3x}\right)-\left(k_{2x}-k_{3x}\right)e^{2i\phi}}{\left(k_{2x}+k_{3x}\right)+\left(k_{2x}-k_{3x}\right)e^{2i\phi}}} \end{aligned} \tag{S11}$$

As before, we want the last line of Eq. (S11) to be expressed in the same form of the Fresnel equation,

$$r^{(s)} = \frac{k_{1x} - k_{eff,x}^{(s)}}{k_{1x} + k_{eff,x}^{(s)}}, \tag{S12}$$

where the effective wavevector component is given by

$$
\begin{aligned}
k_{eff,x}^{(s)} &= k_{2x}\frac{\left(k_{2x}+k_{3x}\right)-\left(k_{2x}-k_{3x}\right)e^{2i\phi}}{\left(k_{2x}+k_{3x}\right)+\left(k_{2x}-k_{3x}\right)e^{2i\phi}} \\
&= k_{2x}\frac{1-\left(\dfrac{k_{2x}-k_{3x}}{k_{2x}+k_{3x}}\right)e^{2i\phi}}{1+\left(\dfrac{k_{2x}-k_{3x}}{k_{2x}+k_{3x}}\right)e^{2i\phi}} \\
&= k_{2x}\frac{1-r_{23}^{(s)}e^{2i\phi}}{1+r_{23}^{(s)}e^{2i\phi}} \\
&\equiv k_{2x}\beta^{(s)}
\end{aligned}
\quad . \tag{S13}
$$

Using the relations, $k_{eff}^{(s)} = n_{eff}^{(s)} k_0 \cos\theta_{eff}^{(s)}$, $\cos\theta_{eff}^{(s)} = \sqrt{1-\left(n_1 / n_{eff}^{(s)}\right)^2 \sin^2\theta_1}$, and $k_{2x} = n_2 k_0 \cos\theta_2$, Eq. (S13) becomes

$$
n_{eff}^{(s)}\sqrt{1-\left(n_1 / n_{eff}^{(s)}\right)^2 \sin^2\theta_1} = n_2 \cos\theta_2 \beta^{(s)} \tag{S14}
$$

Solving Eq. (S14) for $\left(n_{eff}^{(s)}\right)^2$, we can obtain Eq. (2) in the main text.

$$
\begin{aligned}
n_{eff}^{(s)} &= n_1^2 \sin^2\theta_1 + n_2^2 \cos^2\theta_2 \left(\beta^{(s)}\right)^2 \\
&= n_1^2 \sin^2\theta_1 + \left(n_2^2 - n_1^2 \sin^2\theta_1\right)\left(\beta^{(s)}\right)^2
\end{aligned} \tag{S15}
$$

2. Derivation of reflection ratio contrast (Eq. (4))

In this note, we derive the reflection ratio contrast of a 2D layer on the effective substrate, Eq. (4) in the main text. The system of interest consists of the superstrate (*i.e.* the medium 1), the 2D layer (*i.e.* the medium 2), and the reduced substrate (*i.e.* the medium *3'*). We emphasize that we introduce the new subscript *3'* to indicate the reduced substrate whose effective indices are given by Eqs. (1) and (2) in the main text. Since the 2D material is atomically thin, we can Taylor-expand complex reflection ratio $\rho_{2D}(\phi) \equiv r^{(p)}(\phi)/r^{(s)}(\phi)$ up to the first order of the optical path length $\phi = n_2 k_0 \cos\theta_2$ in the 2D layer as follows:

$$\rho_{2D}(\phi) = \rho_{2D}\big|_{\phi=0} + \left.\frac{\partial \rho_{2D}}{\partial \phi}\right|_{\phi=0} \phi = \rho_{sub} + \rho_{sub}\left.\left(\frac{1}{r^{(p)}}\frac{\partial r^{(p)}}{\partial \phi} - \frac{1}{r^{(s)}}\frac{\partial r^{(s)}}{\partial \phi}\right)\right|_{\phi=0} \phi, \tag{S16}$$

where the reflection ratio of the substrate is given by $\rho_{sub} \equiv r_{13'}^{(p)}/r_{13'}^{(s)}$. The reflection coefficient $r^{(p/s)}$ is expressed by the Airy formula, Eq. (S1); it is explicitly written as

$$r^{(p/s)} = \frac{r_{12}^{(p/s)} + r_{23'}^{(p/s)} e^{-2i\phi}}{1 + r_{12}^{(p/s)} r_{23'}^{(p/s)} e^{-2i\phi}} \tag{S17}$$

In Eq. (S17), the Fresnel formula for the interface 23' is given by Eqs. (S2) and (S3), while the effective indices of the reduced substrate are given by Eq. (1) and (2) in the main text. Substituting Eqs. (S17), (S2), (S3) (1), and (2) into Eq. (S16), we can obtain DRCE formulas, Eqs. (4)-(7) in the main text.

3. Determination of the effective index branch in Eq. (1)

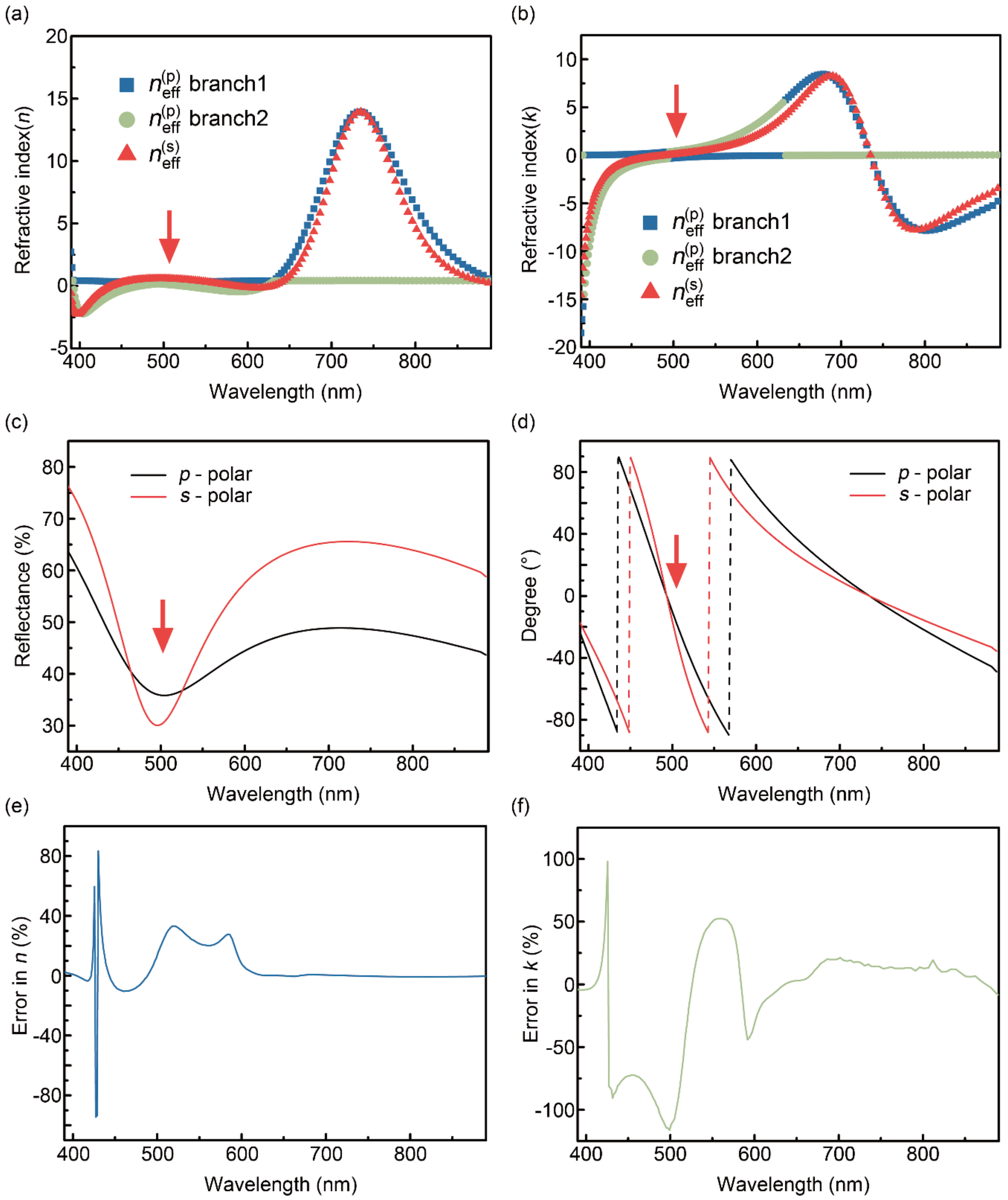

Fig. S1: Effective refractive indices of the reduced substrates including the 280 nm-thick SiO_2 layer and the Si substrate. (a) The real part and (b) the imaginary part of the effective indices at the angle of incidence $\theta_1 = 40°$ (blue squares: plus branch of $n_{eff}^{(p)}$, green squares: minus branch of $n_{eff}^{(p)}$, red triangles: $n_{eff}^{(s)}$). (c) Reflectance, $R_p = |r_p|^2$ and $R_s = |r_s|^2$ (black and red lines: reflectance for the p- and s-polarization, respectively). (d) Phase of the reflection coefficients, $\arg(r_p)$ (black line) and $\arg(r_s)$ (red line). DRCE errors (e) in the real part

($\delta n_{ref} = \left(n_{ref} - n\right) / n_{ref}$) and (f) the imaginary part ($\delta k_{ref} = \left(k_{ref} - k\right) / k_{ref}$) of refractive index of monolayer MoS_2 on the 280 nm-thick SiO_2 layer and the Si substrate. Red arrows in Fig. S1 indicate the spectral region for n_{eff} ~ 0 and DRCE errors.

Effective index of the reduced substrate has ambiguity for the p-polarized light because Eq. (1) has degree of freedom in plus-minus sign although two branches are solutions. However, one can decide the physically meaningful effective index using two criteria: (i) null values of the effective indices are avoided. (ii) The effective indices must be continuous.

Fig. S1a&b show examples of the effective indices of the reduced substrate for the 280 nm-thick SiO_2 layer and the Si substrate. Two branches for the p-polarization have spectral regions for null values, *i.e.* n_{eff} ~ 0. We need to discard branches of null values in the effective index. Interestingly, the resulting effective index $n_{eff}^{(p)}$ show the spectral lineshape similar to $n_{eff}^{(s)}$, except for the bandwidth.

4. Errors in DRCE and null effective index of the reduced substrate

Figs. 2~4 and S1e&f show the error-rich spectral regions in DRCE results. These error-rich regions are related to the interference effect in an optically thick layer. For example, reflectance spectra have dips at the wavelength of ~500 nm in Fig. S1c, while the phases of the reflected light become null at that spectral region in Fig. S1d. This spectral region for the destructive interference in Fig. S1c&d coincides with the null-valued effective index region (Fig. S1a&b) the error-rich region (Fig. S1e&f).

In Eqs. (5) and (6), the functions *A* and *B* vanish for $n_{eff}^{(p/s)}$=0 and ±1. Then, the refractive index of the 2D material layer characterized by DRCE, Eq. (4), becomes indeterminate. Therefore, DRCE errors emerge when the effective indices cross near zero or the unity (the red arrows in Fig. S1a&b).

4. Experiment data for different angles of incidence (AOI)

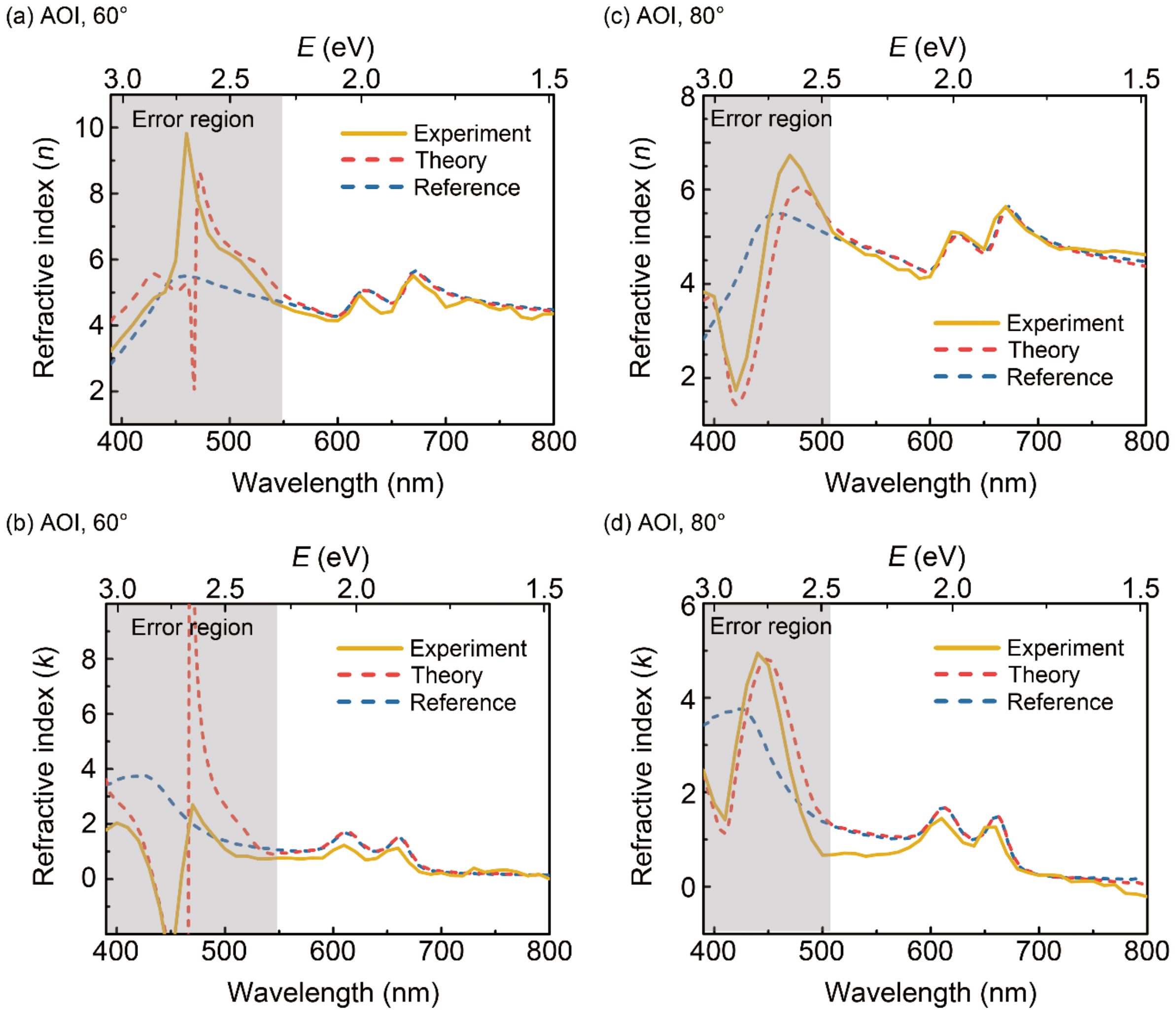

Fig. S2: The refractive index of monolayer MoS_2 on the SiO_2/Si substrate of the oxide thickness 280 nm. (a, c) Real (*n*) and (b, d) imaginary parts (*k*) were obtained at the angle of incidence (AOI) of 60° and 80°, respectively (solid orange lines: DRCE experiment results, dashed red lines: DRCE simulation results, dashed blue lines: reference results taken from [20]).

4. Comparison to conventional techniques

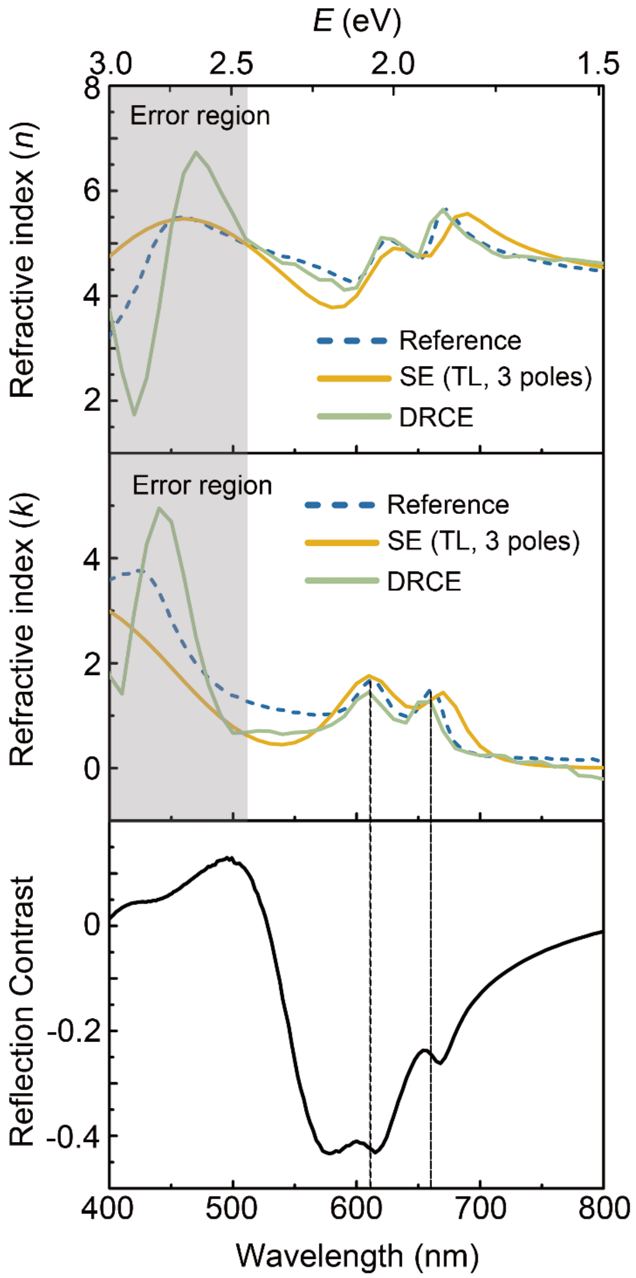

Fig. S3: Comparison of DRCE results (solid green lines), SE results (solid orange lines), and RC spectroscopy results (solid black line in the bottom) for monolayer MoS_2 on a SiO_2/Si substrate (the oxide thickness of 280 nm). Dashed black lines show the wavelengths corresponding to A and B exciton energies. In SE results, the Tauc-Lorentz (TL) oscillator model with three poles is used to fit the refractive index.

4. DRCE simulations for graphene and hBN

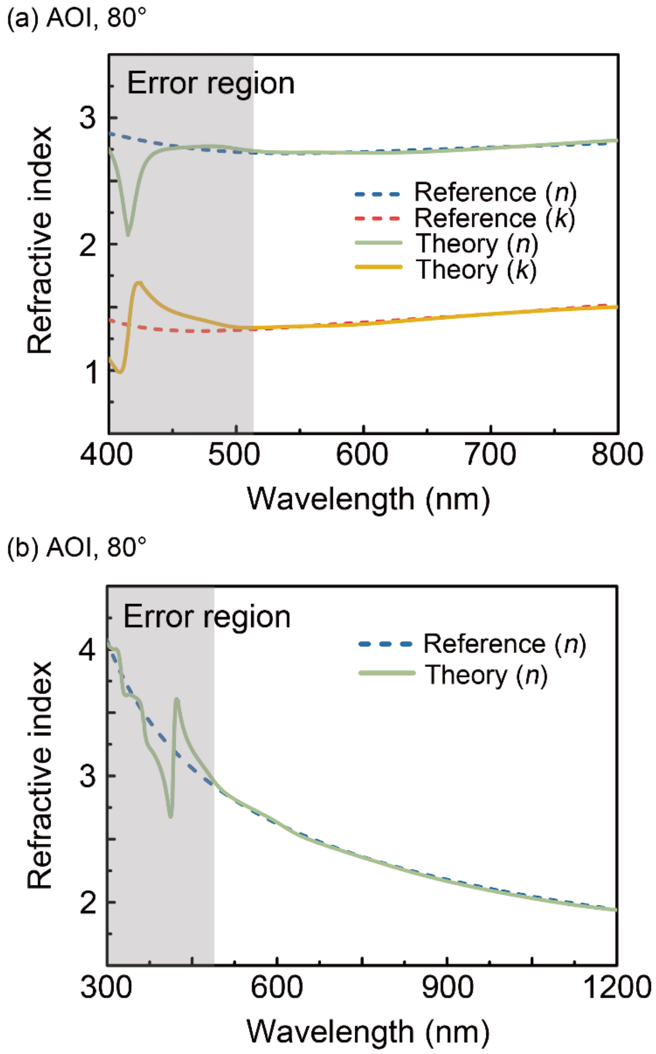

Fig. S4: DRCE simulation results for monolayers (a) graphene and (b) hexagonal boron nitrides (hBNs) on the SiO_2/Si substrate of the oxide thickness 280 nm. Angle of incidence (AOI) is chosen to be 80°.
